# Clinical Impact of HPV Self-Sampling and Molecular Biomarkers on Cervical Cancer Screening and Triage: “The Times They Are A-Changin’”—A Comprehensive Review and Future Perspectives

**DOI:** 10.3390/cancers18162576

**Published:** 2026-08-11

**Authors:** Carlo Liverani, Veronica Boero, Ermelinda Monti, Carlotta Caia, Leonardo Natalini, Paolo Vercellini, Michele Vignali, Andrea Ciavattini

**Affiliations:** 1 Gynecology Unit, Fondazione IRCCS Ca’ Granda Ospedale Maggiore Policlinico, 20122 Milan, Italy; dr.carlo.liverani@gmail.com (C.L.); veronica.boero@policlinico.mi.it (V.B.); ermelinda.monti@policlinico.mi.it (E.M.); carlotta.caia@unimi.it (C.C.); michele.vignali@unimi.it (M.V.); 2 Department of Clinical Sciences and Community Health, University of Milan, 20122 Milan, Italy; 3 Obstetrics and Gynecologic Section, Department of Odontostomatologic and Specialized Clinical Sciences, Università Politecnica delle Marche, 60126 Ancona, Italy; l.natalini@pm.univpm.it; 4 Academic Center for Research on Adenomyosis and Endometriosis, Department of Clinical Sciences and Community Health, Università degli Studi di Milano, 20122 Milan, Italy; paolo.vercellini@unimi.it; 5 Department of Biomedical Sciences for Health, University of Milan, 20133 Milan, Italy

**Keywords:** HPV screening, self-sampling, DNA methylation, exosomes, artificial intelligence, risk-based screening, overtreatment

## Abstract

Human papillomavirus (HPV) infection is a necessary but insufficient cause of cervical cancer (CC) and reflects a long-standing host–virus equilibrium shaped by immune control, viral persistence, and latency. The transition from cytology-based screening to primary high-risk HPV testing has substantially improved early detection of cervical precancerous lesions but has also introduced new clinical challenges related to fluctuating test results, overdiagnosis, overtreatment, and patient anxiety, whose magnitude may vary across countries depending on screening organization and access to prevention services. This narrative review provides a clinically oriented overview of HPV-based CC screening in the context of evolving knowledge on HPV natural history, persistence, and immune interaction, and discusses the implications for the interpretation of test results and risk stratification. New emerging screening and triage approaches, including molecular biomarkers, exosome-based biomarkers, and artificial-intelligence-supported risk assessment, are reshaping CC prevention strategies by enabling more precise identification of women at risk for clinically significant disease. These tools may be particularly relevant in self-sampling-based screening pathways, where molecular triage strategies can reduce the need for additional clinician-collected samples and improve management of HPV-positive women. At the same time, improved understanding of viral latency and host-related determinants of progression supports a shift from binary test interpretation toward longitudinal and individualized risk assessment. Integrating biological insight with technological innovation may facilitate personalized screening strategies that maintain high sensitivity while reducing unnecessary interventions. An evolutionary-informed and risk-adapted approach to HPV-related disease management is essential to optimize prevention outcomes and preserve the benefits of population-based screening programs.

## Simple Summary

Human papillomavirus (HPV) infection is very common and usually clears on its own without causing health problems. However, in some women the virus can persist for many years and increase the risk of cervical cancer. HPV testing is now widely used for cervical cancer screening because it can identify women at risk earlier than traditional Pap tests. At the same time, HPV results can sometimes be difficult to interpret and may cause unnecessary anxiety, testing, or treatment. This review explains how a better understanding of HPV infection, persistence, and immune control can help improve patient management. It also describes the clinical impact of HPV self-sampling and new technologies, including DNA methylation markers, exosome-based biomarkers, and artificial intelligence, that may help identify which women are truly at risk of developing significant disease. These advances could make cervical cancer prevention more accurate, personalized, and less burdensome for patients.

## 1. Introduction

Human papillomaviruses (HPVs) are ancient viral entities that have co-evolved with hominins for hundreds of thousands of years. Genomic and phylogenetic analyses indicate that several high-risk HPV (hrHPV) genotypes—most notably HPV16—diverged from a common ancestor long before the emergence of *Homo sapiens*, with circulation documented in *Homo erectus* and Neanderthal populations. Molecular clock estimates suggest that HPV16 variants have persisted within hominin lineages for approximately 400,000–800,000 years [1].

This evolutionary perspective reframes HPV as a stable component of the human virome rather than a modern pathogen or a consequence of recent lifestyle changes. Long-term, frequently asymptomatic host–virus interactions reflect reciprocal adaptation favoring viral persistence and immune tolerance. Accordingly, HPV infection should be interpreted within an evolutionary and ecological framework, rather than as an immediate clinical threat in all cases. Continuous exposure to HPV antigens over evolutionary time likely contributed to immune modulation and tolerance [2].

From an oncological perspective, the deep evolutionary entrenchment of hrHPV types—particularly HPV16 and HPV18—helps explain their ability to evade immune surveillance, establish persistent infection, and, in specific circumstances, integrate into the host genome. Prolonged co-evolution with human epithelial cells has refined viral mechanisms of latency and reactivation and, in a minority of cases, malignant transformation, contributing to cervical and other anogenital and oropharyngeal cancers.

Given this evolutionary persistence and widespread prevalence, HPV eradication is neither realistic nor biologically plausible. Public health efforts should therefore prioritize reducing oncogenic risk through vaccination, effective screening, and early detection.

A parallel evolution has taken place on the diagnostic and preventive side, as HPV-based screening progressively integrates molecular tools that move beyond binary genotype detection. HPV self-sampling, in which women collect a vaginal specimen autonomously rather than undergoing a clinician-performed cervical sample, has shown diagnostic accuracy for hrHPV detection comparable to clinician-collected samples and is increasingly proposed as an alternative, non-primary collection modality within organized screening programs, particularly to reach under-screened populations [3,4,5]. Because hrHPV positivity alone has limited specificity for clinically relevant disease, triage strategies compatible with self-collected material are needed: host and viral DNA methylation markers have emerged as promising molecular tools able to distinguish transient infections from lesions at risk of progression [6,7]. More recently, exosome-based liquid biopsy, exploiting extracellular vesicles carrying viral and tumor-derived molecular cargo, has been explored as an additional, still investigational, non-invasive approach to cervical cancer detection and monitoring [8,9]. These converging developments in collection modality and molecular triage form the rationale for the present review.

Several recent reviews have specifically addressed HPV self-sampling, focusing on its diagnostic accuracy, acceptability, and cost-effectiveness relative to clinician-collected sampling [10,11,12]. With the performance of self-collection now largely established, the more pressing question concerns the management of HPV-positive women identified through self-collected specimens, namely how they should be interpreted, triaged, and followed over time. The aim of this review is therefore to provide an updated and clinically oriented overview of HPV-based cervical cancer (CC) screening in the context of the evolving understanding of HPV biology, focusing on how emerging tools, including DNA methylation markers and exosome-based signals, can support the interpretation, triage, and follow-up of HPV-positive women identified through self-sampling, while improving risk-adapted and personalized prevention and minimizing unnecessary interventions.

## 2. Materials and Methods

This narrative review is based on a structured literature search of PubMed/MEDLINE, Scopus, and Web of Science, with no language restriction. Search terms combined controlled vocabulary and free-text keywords including “human papillomavirus”, “cervical cancer screening”, “self-sampling”, “self-collection”, “DNA methylation”, “molecular triage”, “exosomes”, “extracellular vesicles”, “artificial intelligence”, and “risk stratification”. Reference lists of retrieved articles and current international guidelines were hand-searched for additional sources. Priority was given to randomized trials, population-based cohorts, systematic reviews, meta-analyses, and guideline documents.

Given the narrative and interpretative scope of this work, a formal systematic review protocol was not applied; the search strategy is reported for transparency rather than as a reproducible systematic methodology.

## 3. HPV and Cancer Burden

High-risk HPV genotypes—particularly HPV16, 18, 31, 33, 45, 52, and 58—cause over 90% of CCs worldwide and contribute to malignancies of the vagina, vulva, anus, penis, and oropharynx [13,14]. Carcinogenesis is driven by the viral oncoproteins E6 and E7, which inactivate p53 and pRb, leading to genomic instability and cellular immortalization, especially in the setting of persistent infection and inadequate immune clearance [15].

Over recent decades, CC incidence has declined, particularly in high-income countries, largely due to organized screening programs and the introduction of prophylactic HPV vaccination. Vaccines initially targeting HPV16 and HPV18, later expanded to include additional oncogenic genotypes (HPV31, 33, 45, 52, and 58), have demonstrated high efficacy in reducing high-grade cervical intraepithelial neoplasia (CIN2/3) and CC in vaccinated populations [16,17]. This protective effect extends to glandular precursors: vaccine efficacy against cervical adenocarcinoma in situ (AIS) has been documented in long-term registry-based follow-up of randomized vaccine trials [18]. This is of particular relevance because adenocarcinoma is less effectively detected by cytology-based screening than squamous disease, so that primary prevention through vaccination assumes proportionally greater importance for this histological subtype.

Despite these advances, substantial global disparities persist. The population-level impact of vaccination depends on several factors, including vaccination coverage, age at vaccination, regional implementation strategies, and the vaccination schedule adopted. Recent evidence from the ESCUDDO trial, a randomized study including 20,330 girls aged 12–16 years, showed that a single dose of the bivalent or nonavalent HPV vaccine was noninferior to two doses in preventing HPV16/18 infection over 5 years, supporting the WHO alternative recommendation for a single-dose schedule in immunocompetent individuals [19].

Beyond CC, hrHPV is increasingly implicated in extra-cervical malignancies, including anal, vulvar, vaginal, penile, and oropharyngeal cancers. Notably, HPV-associated oropharyngeal squamous cell carcinoma has surpassed CC as the most common HPV-related malignancy among men in several Western countries [20]. These trends underscore the need for comprehensive prevention strategies combining vaccination, optimized screening, and innovative approaches such as self-sampling and effective triage of positive results across diverse populations throughout a multidisciplinary counseling.

## 4. The HPV16/18-Cervical Cancer Link

Although 12 HPV genotypes are classified as Group 1 carcinogens, the oncogenic burden is markedly asymmetric. In a systematic analysis of 1174 studies including 111,902 HPV-positive invasive CCs, odds ratios ranged from 48.3 (95% CI 45.7–50.9) for HPV16 to 1.4 (95% CI 1.2–1.7) for HPV51 [21]. HPV16 alone causes an estimated 61.7% of all CCs worldwide and HPV18 a further 15.3%, so that the two genotypes together account for 77.0% of CC worldwide [21].

HPV16 and HPV18 are not interchangeable histologically. HPV16 predominates in squamous carcinoma (63.7% vs. 46.4% in adeno-/adenosquamous carcinoma), whereas HPV18 shows the inverse gradient (38.5% vs. 13.2%) [21]. Additional data confirmed HPV18 to be roughly three times more prevalent in adenocarcinoma (45.3%, 95% CI 42.8–47.8) than in squamous tumors, and HPV18 is associated with 38–50% of AIS despite accounting for only about 8% of all high-grade dysplasia [22,23]. Because glandular precursors arise in the endocervical canal, may present with skip lesions and are poorly detected by cytology, HPV18 positivity carries interpretative weight not captured by CIN3+ risk estimates derived largely from squamous endpoints [23].

This clinical hierarchy has a defined molecular substrate. E6 and E7 are the only viral genes consistently retained and expressed in cervical tumors: E6 targets p53 for proteasomal degradation and activates telomerase, while E7 degrades tumor suppressor gene pRb, driving unscheduled S-phase entry with secondary p16^INK4a^ overexpression—the basis of the p16/Ki-67 dual-stain assay [24,25,26,27]. Integration of viral DNA typically disrupts *E1/E2*, abolishing E2-mediated repression of the early promoter and producing constitutive *E6/E7* expression [28].

Two consequences follow for the framework of this review. First, extended genotyping performed on the same nucleic acid extract used for primary screening provides immediate, morphology-independent stratification without additional sampling—an advantage specific to self-collected specimens. Second, because progression is driven by deregulated *E6/E7* expression and by the epigenetic remodeling accompanying integration rather than by viral presence per se, markers that read transformation are conceptually better aligned with the clinical question than genotype detection alone. Genotype establishes the prior probability of disease; transformation markers refine it.

## 5. HPV-Based Screening and Risk Stratification

The transition from cytology-based screening to hrHPV DNA testing has improved sensitivity for detecting high-risk infections and cervical precancerous lesions, enabling earlier intervention [29,30]. Unlike cytology, hrHPV testing identifies oncogenic potential before morphological abnormalities develop, allowing earlier risk assessment and more personalized management; it can also be performed on self-collected samples [31,32,33].

Risk stratification in hrHPV-based screening relies on:

- HPV genotype: HPV16 and HPV18 confer the highest risk, while types such as HPV31, 33, and 45 are associated with intermediate risk [34,35,36];
- Persistence: sustained infection with the same genotype is the strongest predictor of progression to cervical intraepithelial neoplasia grade 2 or higher (CIN2+) [36];
- Host immune status: immunocompromised individuals have significantly increased risks of viral persistence and neoplastic progression [37].

Together, these parameters allow clinicians to stratify patients into low- and high-risk categories and tailor management accordingly, representing a shift away from the uniform “one-size-fits-all” approach of cytology-based screening.

As most HPV infections regress spontaneously, clinical strategies should increasingly emphasize host immune competence and host–virus interactions, rather than viewing HPV solely as the pathogenic “aggressor.” At the local level, the cervicovaginal microenvironment is central to this control: Langerhans cells, intraepithelial CD8+ T lymphocytes, and natural killer cells constitute the ectocervical first line of defense against viral persistence, while a Lactobacillus-dominant microbiome, particularly L. Crispatus, supports HPV clearance through low vaginal pH, lactic acid production, and modulation of local inflammatory tone [38,39]. Whether these local immune parameters should be formally assessed as part of in-depth diagnostic work-up remains an open question. At present, no standardized, or validated assay exists for quantifying cervicovaginal immune competence in routine practice, and neither immune cell profiling nor microbiome characterization is recommended by current guidelines. Their prognostic relevance is nonetheless biologically plausible and could, if prospectively validated, complement virological and epigenetic markers in identifying women whose infection is more likely to persist. Until such evidence is available, immune status is best incorporated qualitatively, through recognition of clinically evident immunosuppression, rather than through dedicated laboratory testing.

Clinical management should therefore integrate immune status alongside viral detection.

This immunologically oriented framework has several implications:

- Follow-up of low-grade lesions: in CIN1 or equivocal cytological abnormalities (ASC-US, LSIL), spontaneous regression is common, particularly in immunocompetent women under 30, and guideline based surveillance remains the standard of care. Interventions supporting immune function—such as behavioral modifications (e.g., smoking cessation), nutritional supplementation (e.g., vitamin A, folate), or immunomodulatory therapies (e.g., imiquimod, therapeutic vaccines)—may facilitate viral clearance [21]. Furthermore, locally targeted strategies aimed at restoring cervicovaginal immune homeostasis, including intravaginal probiotics to re-establish Lactobacillus dominance and topical immunomodulatory agents, represent an emerging adjunctive approach that may further support HPV clearance and reduce lesion persistence in this setting [40]. However, none of these interventions is currently standard of care for HPV persistence or for the management of low-grade abnormalities.
- Management of high-grade lesions: excisional treatment remains standard for CIN2/3; adjunctive immunotherapeutic strategies, including therapeutic HPV vaccines, may enhance efficacy and reduce recurrence, especially in immunocompromised populations. It should be emphasized that therapeutic HPV vaccines are currently investigational and are not approved for clinical use. Phase I/IIa and phase II trials in HPV16-positive high-grade CIN have reported histological regression and viral clearance in a proportion of treated women, with lesion response correlating with vaccine-induced T-cell responses, but confirmatory phase III data and regulatory approval are still lacking [41,42];
- Post-treatment surveillance: recurrence risk depends not only on surgical margins and post-treatment HPV persistence, but also on the immunological environment; incorporating immune-related factors may enable more individualized follow-up.

This immunologically centered perspective recognizes HPV infection as a necessary but insufficient cause of cancer, with progression driven by dynamic host–virus interactions. Future prevention strategies should therefore aim not only to detect HPV-infected cells but also to support immune mechanisms capable of clearing infection before malignant transformation. Ongoing research into immune profiling, host biomarkers, and immune-based triage tools will be essential to fully realize this paradigm shift in CC prevention.

Table 1 summarizes the current CC screening recommendations issued by the World Health Organization (WHO) [43,44], the American Society for Colposcopy and Cervical Pathology (ASCCP) [45,46], the American Cancer Society (ACS) [4,47], and the European Society of Gynaecological Oncology/European Federation for Colposcopy (ESGO/EFC) [48].

## 6. Interpreting HPV Test Results and Viral Latency

The interpretation of HPV test results is closely linked to the natural history of infection. Epidemiological data indicate that 70–80% of sexually active women acquire at least one hrHPV infection in their lifetime. Most infections are transient and become undetectable within 1–2 years without progressing to clinically relevant lesions, a process traditionally described as “viral clearance” [49,50,51].

However, growing evidence suggests that clearance does not necessarily imply complete viral eradication. HPV can enter a state of virological latency, with immune-mediated control of viral replication. Viral genomes may persist in basal epithelial cells at levels below current assay detection limits, without active replication, genomic integration, or cytological abnormalities.

Support for HPV latency comes from longitudinal studies showing re-detection of identical genotypes after periods of test negativity, particularly with immune senescence, immunosuppression, or other immune modulation [50]. This complicates differentiation between true reinfection and reactivation. Current HPV assays, while sensitive, have defined detection thresholds, so a “not detected” result indicates viral DNA below the assay cutoff rather than absolute absence.

Clinically relevant patterns include:

- Negative HPV tests do not exclude latent or low-level chronic infection;
- HPV re-detection may occur years after apparent clearance, especially if immunity is altered;
- Intermittent positivity may reflect biological fluctuation rather than new exposure.

These observations highlight the need to interpret HPV results within a dynamic host–virus framework. Re-detection of the same genotype is more likely reactivation than reinfection [20]. Women testing “HPV not detected” may still harbor latent or low-copy viral genomes that remain biologically relevant. Most sexually active women are exposed to hrHPV, and latent infections are not reliably detected, suggesting a substantial proportion currently carry or have previously harbored hrHPV [28,32,50,52,53]. Even after serological clearance or negative follow-up tests, molecular or epigenetic traces may persist with potential long-term consequences [15].

Consequently, a negative HPV test does not always equate to uniformly low risk. Prior hrHPV exposure—detectable or not—is associated with increased long-term risk of CIN2+ or CC. Longitudinal studies show higher incidence of CIN2+ in women who test HPV-negative after a previous positive than in women consistently HPV-negative [20,22,23,24,25,26,28,50,52,53,54].

Current guidelines often recommend 5-year screening intervals for HPV-negative women, based on high negative predictive value. However, this may not provide equal protection for all, particularly those with prior exposure. Residual molecular or epigenetic changes may persist, and latent infections can reactivate with age-related immune senescence, new sexual exposures, or physiological stress.

Rather than relying solely on “HPV detected” versus “not detected,” follow-up should consider longitudinal viral history and host-related risk factors. Personalized screening—integrating prior results, immune status, behavior, and potential host or viral biomarkers—could improve risk stratification and reduce interval cancers.

## 7. Managing Fluctuating HPV Results and Avoiding Harm

With widespread HPV-based CC screening, clinicians increasingly encounter temporal variability in test results. Transitions from HPV-positive to HPV-negative status—and occasional reversion to positivity—reflect the natural history of transient, controlled, or latent infection, generating clinical uncertainty and psychosocial burden for patients, partners, and healthcare professionals [28,50,55,56,57].

HPV detection, as a sexually transmitted infection, often triggers anxiety, stigma, and mistrust, particularly when results fluctuate. A negative result after prior positivity may reassure but can also raise questions about viral clearance, latency, immune control, and potential reactivation. Patients frequently seek clarification on changing results, transmission risk, and cancer implications, requiring clinicians to balance scientific nuance, evidence-based caution, and clear communication without providing unwarranted reassurance or alarm.

Fluctuating results may also create interpersonal tension within intimate relationships, amplified by inadequate counseling or insufficient explanation of HPV biology, natural history, and screening principles [58,59,60,61]. In response, both patients and clinicians may favor intensified surveillance; however, excessive testing is often counterproductive. Overreaction can lead to:

- Overtesting: repeated HPV testing or reflex cytology outside guidelines, increasing false positives, costs, and unnecessary interventions;
- Overdiagnosis: detection of transient or clinically insignificant abnormalities (e.g., LSIL or CIN1), resulting in unnecessary disease labeling despite high likelihood of spontaneous regression;
- Overtreatment: interventions for low-grade lesions that expose patients to avoidable morbidity [57].

Although motivated by precaution, this cascade may convert effective population-level screening into a source of iatrogenic harm, emotional distress, and inefficient resource use.

Fluctuating HPV results underscore the need for clinical restraint and contextualized decision-making rather than reflexive escalation. Guideline-based management emphasizing type-specific persistence, individualized risk stratification, and appropriate triage should be consistently applied. Clinician training and patient education must reinforce that transient HPV positivity is common and often benign, recommended surveillance intervals are evidence-based, and not all abnormalities require immediate intervention.

Looking forward, personalized, biomarker-informed screening strategies may reduce reliance on rigid time-based algorithms and limitations of a one-size-fits-all approach. Until such models are implemented, minimizing overtesting and overtreatment is essential to preserve the benefits of molecular HPV screening while avoiding unintended harms.

## 8. Extra-Cervical HPV Infections and Counseling

HPV-related anal and oropharyngeal cancers are increasing, particularly among men who have sex with men (MSM), HIV-positive individuals, and immunosuppressed populations. These trends reflect sexual behaviors, especially orogenital and anal contact, which facilitate hrHPV transmission beyond the cervix [20,62,63,64].

Epidemiological data over the past two decades indicate:

- Anal cancer incidence has risen, especially among MSM, HIV-positive, and immunosuppressed individuals;
- HPV-positive oropharyngeal squamous cell carcinoma (OPSCC) is now the most common HPV-related cancer among men in high-income countries, surpassing CC;
- Anal and oropharyngeal mucosa are highly susceptible to oncogenic HPV types, particularly HPV16, due to epithelial transition zones and relative immune privilege.

Despite these trends, no validated population-based screening programs exist for non-cervical sites. Current HPV screening strategies target CC and are not recommended or validated for anal, oral, or penile cancer in the general population.

Unnecessary testing—such as off-label HPV testing of oral or anal samples without clinical indication—may cause psychological distress and lead to unwarranted procedures [65]. Lack of standardized management guidelines for anal and oropharyngeal HPV infections further increases inconsistent clinical responses and inefficient use of healthcare resources [61,66].

The establishment of International HPV Awareness Day in 2018, celebrated annually on 4 March and supported by organizations such as the ASCCP and the International Papillomavirus Society, represents an important step toward global education.

However, communication strategies matter. Early promotional materials included the message: “Love is everywhere. Unfortunately, so is HPV.” While factually correct, this framing is negatively charged and may inadvertently reinforce negative perceptions of infection.

A more constructive message—such as “HPV is everywhere. Fortunately, love is stronger”—acknowledges viral ubiquity while emphasizing empowerment through vaccination, safe sexual practices, mutual care within couples, and adherence to screening programs. Positive framing can improve acceptance and engagement without minimizing risk.

Effective counseling should normalize HPV exposure, emphasize its typically benign natural history in immunocompetent individuals, discourage unindicated screening, and avoid alarmist messaging, especially in asymptomatic people without known risk factors. This approach prevents psychological harm and preserves the credibility of screening programs [62,63,64,67].

## 9. Triaging HPV Positive Test Results

### 9.1. The Triage Challenge

As HPV testing becomes central to CC screening, managing HPV-positive women presents increasing clinical and organizational challenges. Due to limited specificity for high-grade disease, effective triage is essential to identify women at genuine risk while minimizing unnecessary colposcopy [45,47,68,69,70,71,72].

### 9.2. Cytology and the Self-Sampling Mismatch

Traditionally, cytology has served as the standard triage after a positive HPV result. However, the rise of self-sampling—which improves participation, especially among under-screened populations—has exposed limitations in cytology-based pathways [5,73,74,75,76].

Self-collected vaginal samples detect HPV effectively but do not adequately sample the cervical transformation zone, where high-grade lesions often arise. Consequently, women testing HPV-positive via self-sampling must return for clinician-collected samples, potentially reducing adherence, delaying diagnosis, and diminishing screening gains [3,4,77,78].

The diagnostic performance of self-collection is now well characterized. In the randomized IMPROVE trial, HPV testing on self-collected cervicovaginal samples was non-inferior to clinician collection, with a relative sensitivity of 0.96 (95% CI 0.91–1.03) and relative specificity of 1.00 (95% CI 0.99–1.01) for CIN2+, and corresponding values of 0.99 and 1.00 for CIN3+ [3]. A recent bivariate meta-analysis confirmed these findings across heterogeneous settings, reporting pooled sensitivity of 0.75 and specificity of 0.85 for CIN2+, with self-collection outperforming visual inspection with acetic acid (0.65 vs. 0.54) and showing sensitivity comparable to conventional cytology (0.74 vs. 0.67); agreement with clinician-collected specimens was substantial (kappa = 0.67), and brush-based devices achieved the highest sensitivity among collection tools (0.78) [10].

These favorable estimates should nonetheless be interpreted with caution, since aggregate performance conceals substantial heterogeneity in how self-collection is implemented. Collection devices are not interchangeable: brush-based tools achieved the highest sensitivity for CIN2+ (0.78), whereas agreement between self- and clinician-collected specimens across devices was substantial rather than near-perfect (kappa = 0.67), indicating that a proportion of paired samples yields discordant results [10]. Sampling adequacy depends on the woman herself, and variability in technique, insertion depth, and specimen volume cannot be verified in the laboratory as it can for clinician-collected material. These considerations explain why clinician-collected specimens remain preferred, why self-collection is positioned as an acceptable alternative rather than an equivalent option, and why a shorter three-year retest interval is currently recommended after a negative self-collected test [4].

These considerations are reflected in current screening recommendations. Clinician-collected cervical specimens are preferred and self-collected vaginal specimens are acceptable for primary HPV screening of asymptomatic average-risk individuals. Following a negative primary HPV test, repeat screening is recommended after 3 years for self-collected vaginal specimens, whereas a 5-year interval is recommended for clinician-collected cervical specimens [4].

### 9.3. Extended HPV Genotyping

These challenges highlight the need for molecular triage applicable to self-collected specimens. Extended HPV genotyping represents the most immediately available option, since it can be performed on the same nucleic acid extract used for primary screening and requires no additional sampling [78,79]. By discriminating HPV16 and HPV18 from other high-risk genotypes, and by further stratifying intermediate-risk types, genotyping allows risk-adapted referral without recourse to morphological assessment.

### 9.4. Dual p16/Ki-67 Immunostaining

Dual p16/Ki-67 immunostaining provides a second, morphology-independent triage option [80,81,82,83,84].

In the STAIN study, dual-stained cytology positivity increased progressively with histological severity, from 30.6% in normal histology to 41.5% in CIN1, 72.7% in CIN2, 86.8% in CIN3 and 87.5% in cancer. Compared with HPV testing, dual staining showed lower sensitivity for CIN2+ (80.7%, 95% CI 75.0–85.6 vs. 89.9%, 95% CI 85.3–93.5) and CIN3+ (86.8%, 95% CI 79.7–92.1 vs. 92.3%, 95% CI 86.2–96.2), but higher specificity (64.0%, 95% CI 57.9–69.8 vs. 56.1%, 95% CI 49.8–62.1 for CIN2+), corresponding to a specificity gain of 7.9% for CIN2+ and 9.6% for CIN3+ [81]. Consistently, triage of HPV-positive women with dual staining has been shown to reduce colposcopy referral while maintaining detection of precancerous lesions comparable to cytology-based triage [84]. Its principal constraint in the context of self-sampling is that it remains a cell-based assay requiring adequate cellular material from the transformation zone.

### 9.5. DNA Methylation Markers

DNA methylation analysis has emerged as the most promising option for self-collected specimens, detecting epigenetic changes in viral (*L1/L2* of HPV16/18) and host genes (*CADM1, EPB41L3, FAM19A4, MAL, miR-124, PAX1, SOX1*) associated with CIN2+ and invasive cancer. Methylation assays are objective, reproducible, high-throughput, and suitable for population screening [7,85,86,87,88,89,90].

**Figure 1:**
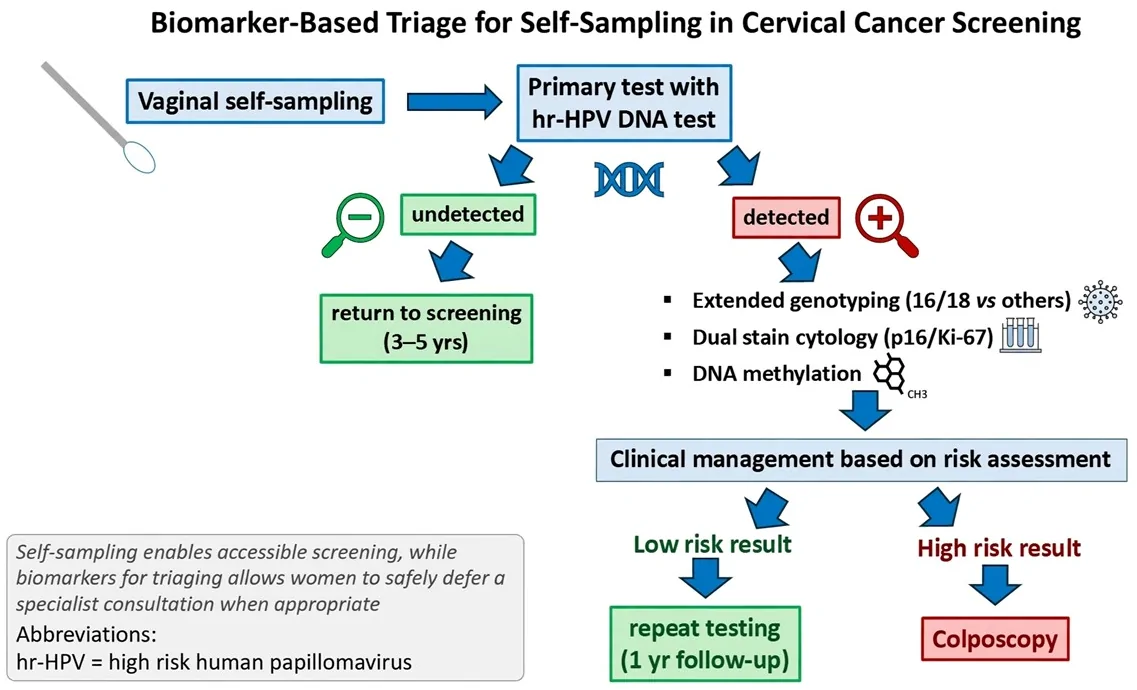
Schematic representation of biomarker-based triage for self-sampling.

The clinical performance of methylation-based triage has been quantified in large multicenter datasets. In the VALID-SCREEN study, which evaluated the *FAM19A4/miR124-2* test on 2384 HPV-positive screening samples from four European countries, sensitivity was 95% for cervical cancer (19 of 20 screen-detected cancers) and 77% for CIN3 (95% CI 71–82), with an overall specificity of 78.3% (95% CI 76–80). Negative predictive values in HPV-positive, methylation-negative women were 99.9% for cervical cancer (95% CI 99.6–99.99), 96.9% for CIN3 or worse (95% CI 96–98) and 93.0% for CIN2 or worse (95% CI 92–94); notably, CIN3 sensitivity was uniform across centers irrespective of collection medium, DNA extraction method or HPV assay [91]. At the meta-analytic level, pooling 43 studies and 16,336 women evaluating host genes (*CADM1, MAL, miR-124-2, FAM19A4, POU4F3, EPB41L3, PAX1, SOX1*) and HPV16 *L1/L2*, methylation achieved a sensitivity of 68.6% (95% CI 62.9–73.8) for CIN2+ and 71.1% (95% CI 65.7–76.0) for CIN3+ at a fixed specificity of 70%. Among HPV-positive women, methylation showed higher sensitivity than HPV16/18 genotyping (relative sensitivity 1.22, 95% CI 1.05–1.42) and a trend toward higher specificity than ASC-US cytology (relative specificity 1.25, 95% CI 0.99–1.59) [87]. Biomarker-based triage for self-collected specimens is schematically summarized in Figure 1.

### 9.6. A Two-Step Molecular Strategy for Self-Collected Specimens

A two-step strategy could optimize self-sampling: primary HPV testing on a self-collected sample, followed by reflex methylation testing for hrHPV-positive cases. Methylation-negative women would return to routine screening, while methylation-positive cases would proceed directly to colposcopy. This preserves accessibility, reduces unnecessary referrals, and avoids additional clinical sampling. Methylation assays are compatible with both self- and clinician-collected specimens, allowing seamless integration into existing programs [92,93].

As HPV-based screening expands, methylation-based triage offers a scalable solution to reduce follow-up loss, relieve colposcopy demand, prevent overtreatment, and maintain high sensitivity for clinically significant lesions. By removing reliance on cytology, methylation testing supports a fully molecular, decentralized screening model essential for equitable and effective CC prevention.

## 10. Emerging Directions

### 10.1. Exosomes as Emerging Biomarkers and Therapeutic Tools

Once considered cellular waste carriers, exosomes—a subclass of extracellular vesicles, 30–200 nm in diameter—are now recognized as key mediators of intercellular communication, immune regulation, and oncogenic processes. Released by virtually all cell types, including malignant and virus-infected cells, they are detectable in biological fluids such as blood, urine, saliva, sweat, and vaginal secretions [94].

Exosomes can cross selective biological barriers, including the blood–brain and placental barriers. Their stable lipid bilayer and complex molecular cargo—proteins, lipids, mRNAs, microRNAs (miRNAs), and DNA fragments—reflect the state of the parent cell. In HPV-associated diseases, exosomal content provides a dynamic, real-time readout of viral activity, oncogenic transformation, and therapeutic response [95,96].

In CC and HPV-related head and neck squamous cell carcinoma (HNSCC), exosomes are promising diagnostic biomarkers [97]. Protein and RNA profiles of tumor-derived exosomes correlate with:

- hrHPV genotype and viral transcriptional activity (e.g., E6/E7 mRNA);
- Tumor stage and progression;
- Immune evasion and inflammatory signatures;
- Treatment response and recurrence risk [8].

Non-invasive analysis of exosomes from vaginal secretions or cervicovaginal lavage could, in principle, complement or partially replace cytology and DNA methylation triage, particularly in self-sampling programs, enabling clinician-independent risk stratification.

Beyond diagnostics, tumor-derived exosomes actively promote disease by shaping the tumor microenvironment, suppressing immunity, and enhancing angiogenesis. Targeting exosome release, uptake, or function may offer therapeutic approaches against high-grade lesions and HPV-associated cancers [8,9,98,99,100,101,102,103].

Although clinical application is early, evidence for exosome relevance in HPV-related conditions is rapidly growing. Challenges include standardizing isolation, selecting biomarkers, and ensuring reproducibility. Exosomes are promising dual-purpose tools for diagnostics and therapeutics, bridging self-sampling, molecular triage, and precision medicine. Unlike HPV tests, exosomes reflect tumor biology directly, signaling malignancy independently of HPV status-relevant since 5.5–11% of CCs and 10–15% of high-grade lesions test HPV-negative [100].

Several practical barriers still limit the clinical translation of exosome-based assays. Isolation techniques, ranging from ultracentrifugation to size-exclusion chromatography, immunoaffinity capture, and microfluidic platforms, differ considerably in yield, purity, and cost, and none currently meets all requirements for routine diagnostic use [104]. Detection platforms such as electrochemical and aptamer-based biosensors are promising but remain at a proof-of-concept stage [105]. Importantly, no exosome-based assay has yet been validated in cervicovaginal specimens within a screening population, and none is commercially approved for cervical triage. Their clinical role therefore remains prospective rather than established.

Therapeutically, exosomes can deliver nucleic acids or pharmacological agents with high biocompatibility, low immunogenicity, and cell-specific tropism. In HPV-related diseases, they are being explored as platforms for therapeutic vaccines, targeted immunotherapies, and delivery of chemo- or radiosensitizers. Emerging studies, including work by Cakir et al., support their feasibility while highlighting the need for broader validation [8].

**Figure 2:**
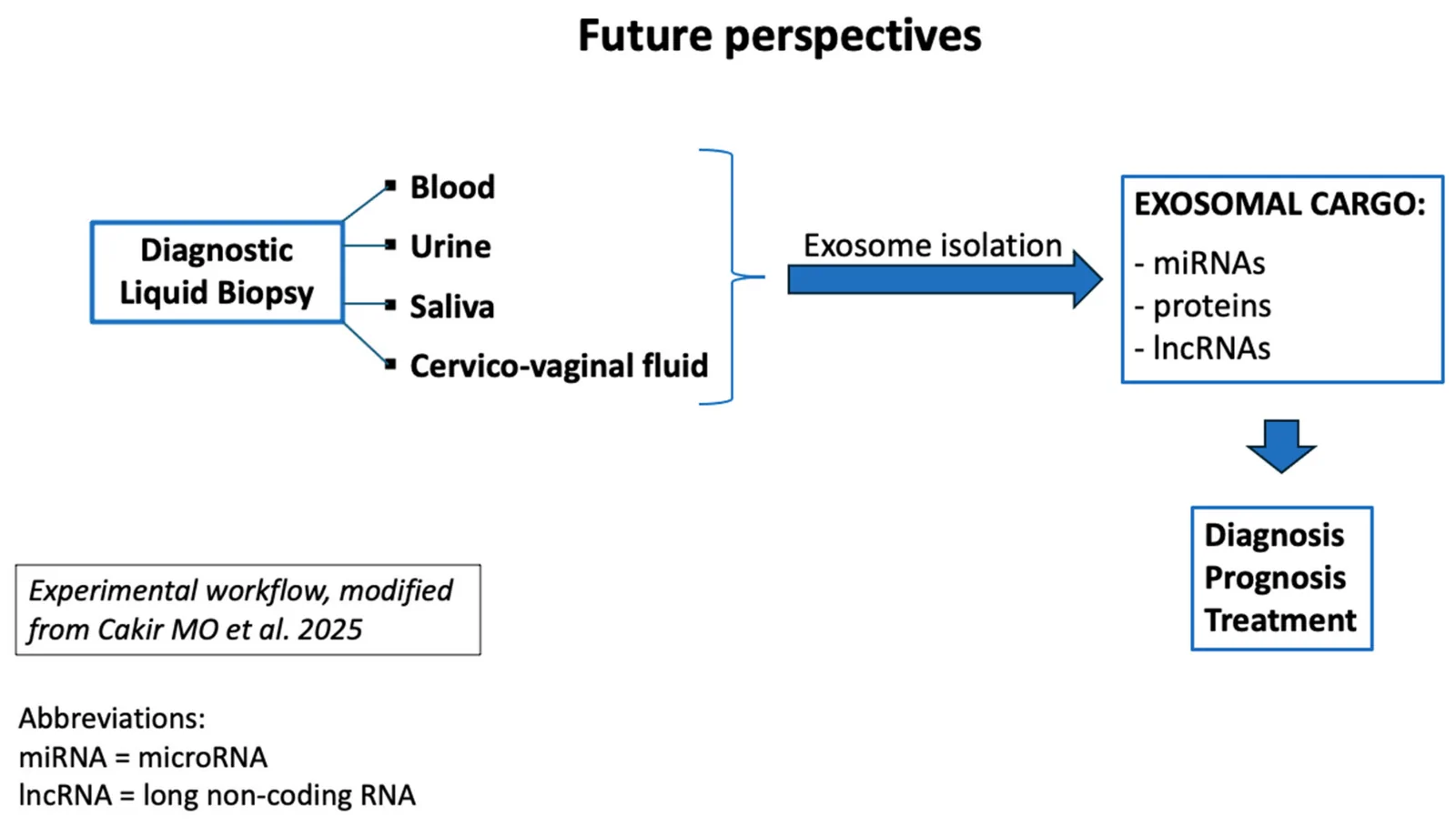
Proposed experimental workflow for exosome-based liquid biopsy in HPV-related cervical disease [8].

A schematic overview of the proposed exosome-based liquid biopsy workflow, from sample collection to potential clinical application, is shown in Figure 2.

### 10.2. Artificial Intelligence (AI) in HPV Screening and Triage

Artificial Intelligence (AI) is rapidly transforming healthcare by enabling data-driven decision making, improved diagnostic accuracy, and more efficient workflows. In CC prevention, AI shows particular promise in triage and post-screening management of HPV-positive individuals [106].

A leading AI application is the PAVE (Primary HPV testing Automated Visual Evaluation) approach, which uses deep learning to analyze cervical images and identify high-grade lesions. Developed through international collaborations, PAVE demonstrated:

- High sensitivity and specificity for CIN2+ lesions;
- Consistent performance across diverse settings, including low-resource environments;
- Potential as an alternative to colposcopy in selected contexts, although no prospective comparison within an organized screening programme has yet been reported.

Combined with primary HPV testing, PAVE and similar AI tools support a “Screen-Triage-Treat” strategy, reducing diagnostic delays, minimizing loss to follow-up, and improving program efficiency [101,102,103,107,108].

Beyond imaging, AI enables individualized risk prediction by integrating HPV results, molecular biomarkers, clinical history, and sociodemographic factors. It can also automate follow-up, optimize patient education, and streamline health system workflows, including scheduling and resource allocation.

By reducing cognitive and administrative burdens, AI allows specialists to focus on complex cases, multidisciplinary decisions, and patient-centered care. Successful implementation requires attention to algorithmic bias, transparency, data security, regulatory approval, and interoperability with existing systems.

It should be emphasized that none of these applications currently forms part of routine cervical screening practice. AI-assisted triage remains at the stage of validation studies and pilot implementations, and its place in the screening pathway will depend on prospective evaluation within organized programmes rather than on retrospective diagnostic accuracy alone.

## 11. Conclusions

HPV eradication is biologically implausible, and prevention must therefore focus on risk control rather than on viral elimination. This review examined the potential applications, advantages, and limitations of HPV self-sampling, and outlined the current state of the art on the molecular biomarkers capable of refining its diagnostic accuracy. Self-collected specimens achieve a diagnostic performance for hrHPV detection comparable to clinician-collected samples when clinically validated PCR-based assays are used, although heterogeneity across collection devices and variability in sampling quality explain why self-collection is currently positioned as an acceptable alternative rather than an equivalent option. Its principal limitation is not analytical but downstream, since the conventional triage pathway remains cytology-dependent and therefore requires an additional clinician-collected sample. Molecular triage, and DNA methylation testing in particular, addresses this constraint directly, as it can be performed on the original self-collected specimen and identifies transforming rather than merely prevalent infection. Binary interpretation of HPV results is no longer sufficient: longitudinal viral history, genotype-specific persistence, and host immune competence should inform risk-adapted management, in order to avoid both undertreatment and iatrogenic harm.

Future research should prioritize the prospective validation of integrated molecular triage strategies on self-collected specimens, the standardization of collection devices and device-assay pairings, and the development of implementation frameworks for risk-adapted screening across diverse healthcare settings. An evolutionary-informed, risk-based, and patient-centered approach remains essential to reduce the global burden of HPV-related cancers while minimizing unnecessary interventions and preserving trust in screening programmes.

## Figures and Tables

**Table 1:** Comparison of current international guidelines on the organization of CC screening.

Parameter	WHO [43,44]	ASCCP [45,46]	ACS [4,47]	ESGO/EFC [48]
**Target population/starting age**	General population: 30 years; women living with HIV: 25 years	No independent starting age; adopts ACS primary screening parameters; management algorithm applies from age 25	25 years (average risk)	No formal consensus on starting age; EU range accepted 20–29 years, not before 25; most protocols target women >30 years
**Recommended** **primary test**	HPV DNA/mRNA test (screen-and-treat or screen-triage-and-treat approach)	Not specified (management-focused guideline); accommodates HPV primary testing, co-testing or cytology per local program	Primary HPV testing (preferred); co-testing or cytology alone acceptable where HPV testing unavailable	Primary HPV testing from age 30; primary cytology retained below age 30 due to high transient-infection prevalence
**Screening interval**	5–10 years (general population); 3–5 years (HIV-positive)	Not applicable to primary screening; post-triage retest intervals risk-stratified (typically 1 or 3 years)	5 years with primary HPV testing	5–7 (10) years with HPV testing vs. 3–5 years with cytology; interval extendable in HPV-negative women
**Self-sampling policy**	Equivalent recommendation for provider-collected and self-collected specimens	Not regulated directly; adopts ACS/Enduring Consensus Committee guidance of 3-year retest after a negative self-collected HPV test	Introduced in the 2025 update: self-collection with FDA-approved devices; 3-year retest interval if negative	Recommended to reach non-attenders/under-screened women; population-wide mailing not currently recommended
**Triage of** **HPV-positive women**	Partial genotyping, colposcopy, VIA, cytology, or (2024) dual-stain p16/Ki-67 cytology	Risk-based algorithm using two distinct thresholds: immediate CIN3+ risk (~4%) determines direct colposcopy/treatment; 5-year CIN3+ risk determines return to 1-year, 3-year, or routine 5-year screening	Defers to ASCCP/Enduring Consensus management guidelines for triage of positive results	Reflex cytology (only triage test with high-grade evidence); genotyping, p16, methylation and viral load under evaluation
**Screening exit criteria**	After age 50, following two consecutive negative results (general population and HIV-positive)	Not specified (outside management-guideline scope)	Negative HPV test or co-test at ages 60 and 65; if unavailable, three consecutive negative cytology tests, last at age 65	Age 65 with a negative exit test; non-attenders may still be invited beyond age 65
**Special populations**	Dedicated algorithm for women living with HIV (start age 25, priority group 25–49 years)	Dedicated algorithms for immunocompromised patients, pregnancy, and post-treatment surveillance	Not addressed in the 2025 update (average-risk guideline only)	No dedicated algorithm for immunocompromised or pregnant patients; substantial dedicated discussion of screening strategies stratified by HPV vaccination status

## Data Availability

No new data were created or analyzed in this study. Data sharing is not applicable to this article.

## Abbreviations

The following abbreviations are used in this manuscript:

AI	Artificial Intelligence
AIS	Adenocarcinoma In Situ
ASC-US	Atypical Squamous Cells of Undetermined Significance
ASCCP	American Society for Colposcopy and Cervical Pathology
BCE	Before Common Era
CC	Cervical Cancer
CIN	Cervical Intraepithelial Neoplasia
HIV	Human Immunodeficiency Virus
HNSCC	Head and Neck Squamous Cell Carcinoma
HPV	Human Papillomavirus
hrHPV	High-risk Human Papillomavirus
HSIL	High-grade Squamous Intraepithelial Lesion
Ki-67	Antigen Kiel 67 (proliferation marker)
LSIL	Low-grade Squamous Intraepithelial Lesion
miRNA	MicroRNA
mRNA	Messenger Ribonucleic Acid
MSM	Men who have Sex with Men
OPSCC	Oropharyngeal Squamous Cell Carcinoma
p53	Tumor Protein 53
PAVE	Primary HPV testing Automated Visual Evaluation
pRb	Retinoblastoma Protein
RNA	Ribonucleic Acid
VIA	Visual Inspection with Acetic acid
